# A targeted next-generation sequencing method for identifying clinically relevant mutation profiles in lung adenocarcinoma

**DOI:** 10.1038/srep22338

**Published:** 2016-03-03

**Authors:** Di Shao, Yongping Lin, Jilong Liu, Liang Wan, Zu Liu, Shaomin Cheng, Lingna Fei, Rongqing Deng, Jian Wang, Xi Chen, Liping Liu, Xia Gu, Wenhua Liang, Ping He, Jun Wang, Mingzhi Ye, Jianxing He

**Affiliations:** 1 BGI-Shenzhen, Shenzhen, 518083, China; 2 Department of Biology, University of Copenhagen, Copenhagen, DK-2200, Denmark; 3 BGI-Guangzhou, Guangzhou Key Laboratory of Cancer Trans-Omics Research, Guangzhou, 510006, China; 4 Department of Laboratory Medicine, The First Affiliated Hospital of Guangzhou Medical University, Guangzhou, 510120, China; 5 Centre of Translational Medicine, The First Affiliated Hospital of Guangzhou Medical University, Guangzhou, 510120, China; 6 The First Affiliated Hospital of Guangzhou Medical University, Guangzhou, 510120, China

## Abstract

Molecular profiling of lung cancer has become essential for prediction of an individual’s response to targeted therapies. Next-generation sequencing (NGS) is a promising technique for routine diagnostics, but has not been sufficiently evaluated in terms of feasibility, reliability, cost and capacity with routine diagnostic formalin-fixed, paraffin-embedded (FFPE) materials. Here, we report the validation and application of a test based on Ion Proton technology for the rapid characterisation of single nucleotide variations (SNVs), short insertions and deletions (InDels), copy number variations (CNVs), and gene rearrangements in 145 genes with FFPE clinical specimens. The validation study, using 61 previously profiled clinical tumour samples, showed a concordance rate of 100% between results obtained by NGS and conventional test platforms. Analysis of tumour cell lines indicated reliable mutation detection in samples with 5% tumour content. Furthermore, application of the panel to 58 clinical cases, identified at least one actionable mutation in 43 cases, 1.4 times the number of actionable alterations detected by current diagnostic tests. We demonstrated that targeted NGS is a cost-effective and rapid platform to detect multiple mutations simultaneously in various genes with high reproducibility and sensitivity.

Lung cancer is the most common cancer and the leading cause of cancer death in China, accounting for 1/5 of total cancer incidence and 1/4 of all cancer-related mortality^1^. The incidence rate of lung cancer in China has been increasing during the past several decades at a more rapid rate than in western countries^1^. The treatment of non-small cell lung cancer (NSCLC) with platinum-based chemotherapy has reached a plateau of effectiveness. Personalised therapy which targets tumour-specific molecular abnormalities is gradually starting to play an important role in the systemic treatment plan for cancer patients^2^. In lung adenocarcinoma, multiple genetic alterations have already been identified as therapeutic targets, including mutations of the *EGFR* gene and rearrangements of the *ALK* and *ROS1* genes. Drugs designed specifically as inhibitors of these molecular targets have significantly extended the survival times for patients whose tumours harbour these mutations^3^,^4^,^5^,^6^. In addition, several other target oncogenes with potential prognostic role in lung adenocarcinoma, including *MET*, *PIK3CA*, and *RET*, have also been described, and target agents are currently under evaluation^7^.

Given the increased availability of various targeted therapies, comprehensive characterisation of mutations in clinically actionable genes and key cancer pathways have become necessary to improve our understanding of the genetic basis of the disease, to choose suitable treatment options, and to aid in the estimation of prognosis and drug resistance^8^,^9^. However, this has also brought a great challenge to routine pathology, as approximately 64% of lung adenocarcinomas harbour at least one somatic oncogenic mutations in *EGFR*, *HER2*, *KRAS*, *PIK3CA*, *BRAF*, *MEK1*, and *ALK*^10^,^11^. In current clinical practice, tumor molecular profiling involves multiple assessments (Amplification Refractory Mutation System PCR, Sanger sequencing, fluorescence in situ hybridisation, and immunohistochemistry), each of which targets a single gene or type of mutation, resulting in increased costs and turnaround time. In addition, when mutational assessment of multiple genes is needed, traditional methods require a large quantity of DNA, which is often difficult to obtain in clinical settings^12^.

Massively parallel or next-generation sequencing technology is increasingly being used for mutational analysis of tumours for both clinical and research applications^13^. Targeted next-generation sequencing of cancer-related genes allows rapid detection of a variety of somatic mutations on single platforms (such as Ion Proton and MiSeq)^16^,^17^,^18^. Although, many studies have demonstrated the feasibility of this method, there are several problems to overcome before it can be applied to routine diagnostics in the clinic^19^,^20^,^21^. Firstly, in routine diagnostics, the majority of cancer specimens are formalin-fixed, paraffin-embedded (FFPE) samples, which yield DNA in limited quantity due to degradation and cross-linking. Therefore, optimised sequencing library preparation for fragment DNA input is required. Secondly, an efficient and robust algorithm is needed to detect all types of cancer mutations, including SNVs, InDels, CNVs, and gene rearrangements. Finally, the NGS method should be comparable with current conventional test in all aspects, including turnaround time, sensitivity, specificity, mutation detection limits, costs, and capacity.

To evaluate the feasibility of applying an NGS technique to mutation analysis of routinely obtained specimens in a clinical molecular diagnostic laboratory, we developed a targeted NGS test designed to detect 436 exons in 145 genes relevant to personalised therapy in lung cancer. Next, we validated the method in retrospective FFPE-DNA samples and cancer cell lines by comparing the results with those obtained by conventional methods. Finally, we assessed the performance of the validated test in routine pathology practice.

## Results

### Description of workflow

**Figure 1:**
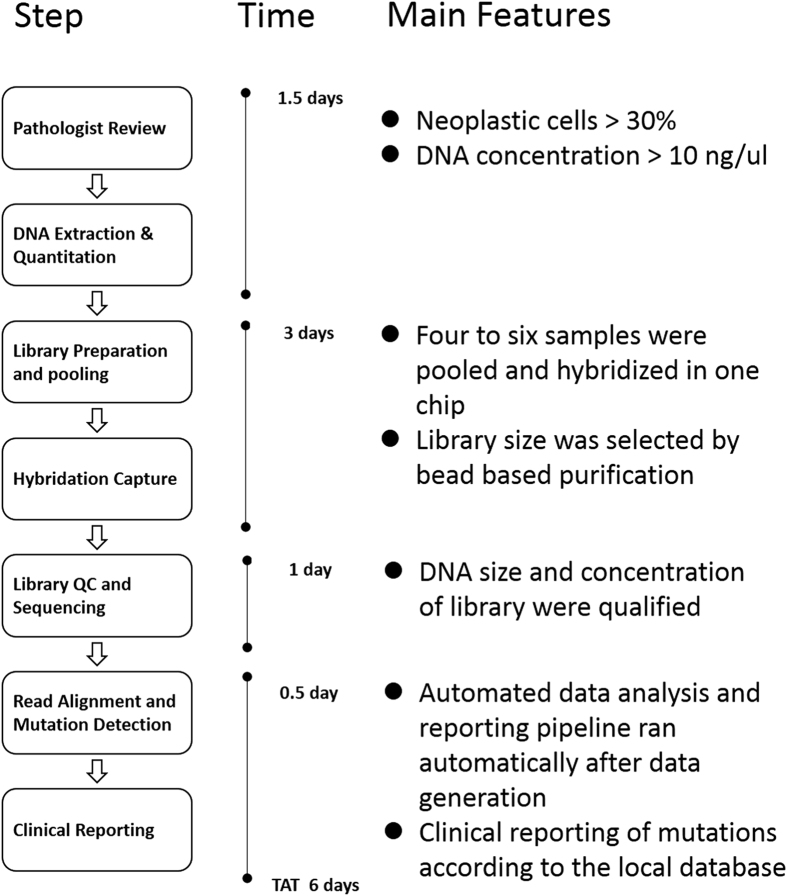
Workflow and turnaround time for molecular profiling of clinical samples using targeted NGS. Abbreviations: QC, Quality Control; FFPE, Formalin-Fixed, Paraffin-Embedded; TAT, turnaround time; NGS, next-generation sequencing.

We developed and optimised a targeted next-generation sequencing technical platform to detect actionable and clinically relevant mutations in clinical routine specimens. An overview of the test is shown in Fig. 1, the entire workflow lasts approximately six days. In brief, following a quality assessment of FFPE samples by a pathologist, quantified DNA was used to prepare the NGS sequencing library, during which a six-base pair DNA barcode was ligated to the ends of DNA fragments, allowing multiple samples to be pooled before hybrid selection. These DNA libraries were subjected to solution-phase hybrid capture with biotinylated RNA baits targeting 436 exons from 145 cancer related genes. After a quality control (QC) step, three captured libraries were pooled and sequenced by the Ion Proton instrument. An average sequencing output of 13.48 gigabases (Gb) was generated for each run. SNVs, InDels, CNVs and gene arrangements were detected by a customised analysis pipeline. Finally, the test report was automatically generated based on the drug and mutation database.

### Hybridisation chip design and capture performance

To reduce genome complexity prior to sequencing, we designed a panel of 145 clinically related genes in lung cancer. These genes were divided into two main parts (Supplementary Table 1). Part I genes harbour mutations that are clinically actionable now or in the future; these include targets of existing targeted therapies in the National Comprehensive Cancer Network (NCCN) guidelines and targets under active development in clinical trials. Part II genes include prognostic markers, and other oncogenes and tumour suppressors that are frequently mutated in cancer according to the Catalogue of Somatic Mutations in Cancer (COSMIC), The Cancer Genome Atlas (TCGA) or the International Cancer Genome Consortium (ICGC). Altogether, these genes comprised 436 exons encoding 249 kilobases. We then designed and synthesised 88,102 unique biotinylated RNA baits corresponding to these genomic regions.

Initially, we established our approach using genomic DNA from cell lines with known mutations. Due to the poor quality of FFPE DNA, we then optimised library preparation and hybridisation methods using archival FFPE samples. In the final protocol, four to six libraries were subjected to a single hybrid reaction, with up to 20 barcoded libraries pooled for sequencing in a single Ion Proton run. This produced 13 Gb of raw data, which corresponds to approximately 6.9 million reads per sample. After marking duplicates and filtering low quality reads, an average of 4.7 million clean reads were generated per sample. The percent of effective bases on target averaged 32.99%, the average depth of the target exceeded 450-fold, and more than 96.26% bases had at least 20-flod coverage, indicating the suitability of this method for identifying mutations (Figure S1).

### Mutation detection limit and reproducibility assessed by cell lines

We designed an analysis program with the theoretical aim of reaching the level of sensitivity and specificity beyond 99% for mutant allelic frequency >0.03. Performance was verified to be reliable by the following procedure. We mixed positive cell lines that harboured actionable mutations, whose allelic frequency was considered as 50%, with DNA from the YH cell line, to make up virtual tumour samples harbouring gradients of hierarchical allelic frequency, i.e. 1%, 3%, 5%, 10%, successively. For SNVs, we detected all six mutations with an allelic frequency down to 3%, and 4/8 (50%) mutations with only 1% allelic frequency. Regarding InDels, all mutations including those with an allelic frequency of 1%, were detected (Table 1). The measured value of the detection limit in the serial dilution studies was in accordance with the theoretical value. Intra-run reproducibility was tested with three samples harbouring three mutations across two genes. Eight replicate libraries with different barcodes from each sample were pooled for hybridisation and sequencing in an Ion Proton run. All the mutations were detected reproducibly, and the depth of aligned reads and detected variant read frequency were highly reproducible (Table 2).

### Sequencing of archival samples with known mutations

We then evaluated the performance of targeted NGS in a clinical setting using FFPE tissue from 61 patients with lung adenocarcinoma. All samples underwent successful targeted sequencing of the 145 genes. We compared the gene mutation results of targeted NGS with the results (including mutations in *EGFR*, *KRAS*, *BRAF*, *PIK3CA*, *ALK*, and *ROS1*) obtained from a variety of current clinical technologies, including amplification refractory mutation system PCR (ARMS-PCR), Fluorescence *in situ* hybridization (FISH) and immunohistochemistry (IHC). A concordance rate of 100% between the results of conventional platforms and NGS were archival (Table 3). All mutations detected by the conventional methods were detected by targeted NGS. Several mutations were detected in samples containing only 10–15% tumour cells, in which the CT value of ARMS-PCR was close to the cut-off value (Table S2). Moreover, additional four mutations in these six genes were identified because of the high sensitivity of targeted NGS. In some cases, a major driver mutation coexisted with other variants, as in the case of #A21, which showed a deletion mutation in *EGFR* EX19 in 17.54% of mutant alleles as well as lower frequency H1047L mutation (8.87%) in *PIK3CA* (Table S2). Furthermore, our pipeline found additional actionable mutations as a result of the broader target range compared with the traditional methods (Figure S2).

### Data interpretation and reporting

To aid accurate interpretation of the data from the assay and the subsequent communication of that information to clinicians, we developed an automatic process for data interpretation and reporting. By aggregating information from publicly available resources such as My Cancer Genome, the Gene-Drug Knowledge Database, the NCCN guidelines, primary literature, and expert opinion, we first generated a database of tumour alterations relevant for personalised lung cancer therapy. The database integrated 115 variants, including 49 SNVs, 45 InDels, 19 fusions and 2 amplifications in 15 genes with 27 targeted drugs. Following the analysis and annotation, a final list of the variants was produced, and the clinical action associated with each variant, and the negative genes for which no mutations were detected. According to an internally established classification scheme, evidence of potential drug efficacy or resistance in lung cancer patients carrying the somatic variants were classified into two categories and with four to five levels, as summarised in Table 4.

### Detection of clinically actionable alterations in patients

**Figure 2:**
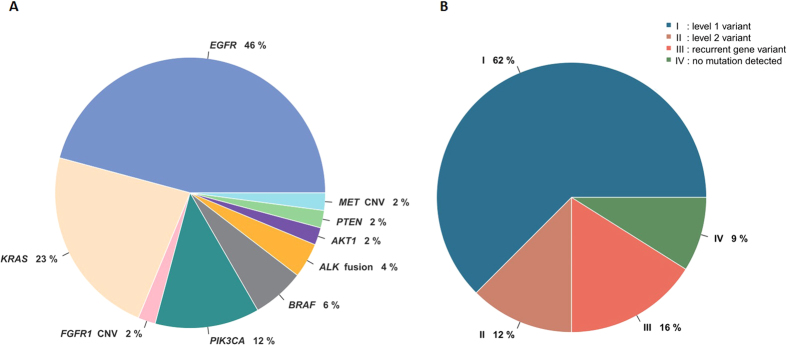
Relative proportions of genomic aberrations in lung adenocarcinoma (N = 58). (**A**) Actionable variants and gene frequencies detected in 58 prospective samples. (**B**) Distribution of the patients according to the classification of detected actionable mutations.

Having demonstrated that the targeted NGS test displayed sufficient to detect multiple mutations with high reproducibility and sensitivity, we further sought to assess the value of practical application of the test in a clinical setting. A total of 58 patients diagnosed with lung adenocarcinoma were included in a prospective study from January 2015 to May 2015. Of the samples for which sequencing was possible, 62% of the samples contained a level 1 variant, twelve percent of the samples had a level 2 variant. By this measure, at least one potentially actionable variant (level 1 or 2) was identified in 74% of the samples that could be sequenced, 1.4 times the number of actionable alterations detected by current diagnostic tests. Figure 2 shows the frequencies of genetic alterations in patients with lung adenocarcinoma. The genetic alterations included: an *EGFR* mutation in twenty two (46%) patients; a *KRAS* mutation in eleven (23%); a *PIK3CA* mutation in six (12%); a *BRAF* mutation in three (6%); an *ALK-EML4* fusion in two (4%); a *MET* copy number gain in one (2%); a *PTEN* mutation in one (2%); an *AKT* mutation in one (2%) and an *FGFR1* copy number gain in one (2%). Of the 22 patients with *EGFR* mutations, 11 contained the L858R point mutation in exon 21, and 10 had deletions in exon 19. There was high concordance between the NGS platform and conventional methods; of the 38 mutations identified by ARMS-PCR or FISH, 36 mutations were also called by NGS. Furthermore, using the targeted NGS platform, additional mutations in other genes were identified in these 58 tumour samples as shown in Figure S2.

## Discussion

**Figure 3:**
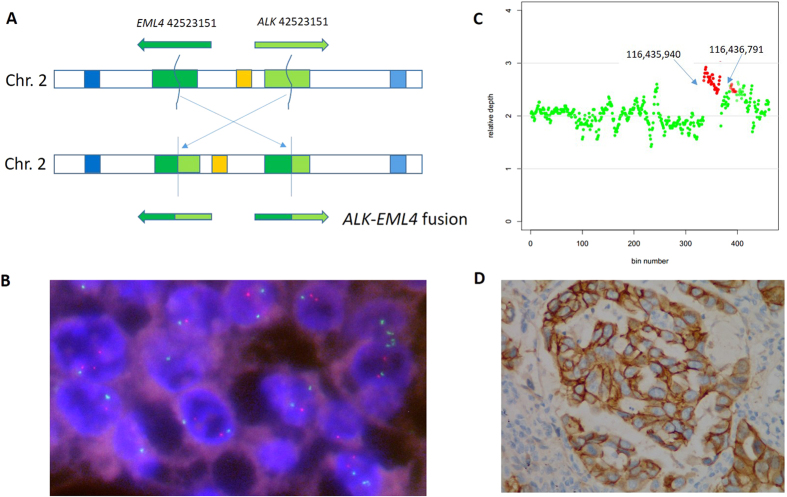
Gene fusion and CNV detected in archival samples. (**A**)*ALK-EML4* fusion found in sample #A59. *ALK* and *EML4* break at chr2 29446561 and chr2 42523152, respectively. Then the minus stand of *EML4* was reverse conjoined to *ALK*. (**B**) Confirmation of *ALK-EML4* fusion by FISH. (**C**) *MET* amplification found in sample #P46. Gene region from 116435940 to 116436719 on chromosome 7 had a copy number of three. (**D**) Confirmation of *MET* amplification by immunohistochemistry.

Multiple genetic alterations have already been identified as therapeutic targets for lung adenocarcinoma. Comprehensive characterisation of mutations in clinically actionable genes and key cancer pathways can be helpful for prognostic prediction and guiding the selection of therapy, ultimately accelerating the development of personalised treatment^2^. To this end, we established an NGS mutation assay for the detection of actionable mutations in routine FFPE samples of lung adenocarcinoma which we have comprehensively demonstrated to perform to a high clinical standard. In this study, we sequenced 145 cancer related genes from 119 FFPE tumour DNA samples. Benefiting from the optimisation of library preparation for FFPE DNA, we archival 480-fold mean coverage per sample. The uniformly high sequence coverage across all test regions afforded robust, simultaneous detection of SNVs, InDels, amplifications, and gene rearrangements (Fig. 3). The overall performance of our test was high. In cell line models, the mutation detection limit was 5% for base substitutions, and 1% for InDels. Compared to current clinical tests, the concordance on mutually tested markers exceeded 95% for all alteration types. In contrast to the commercial multiple PCR panels such as Ion Ampliseq (Life technologies, Carlsbad, CA) which cannot detect gene rearrangement in FFPE DNA, our approach permits the detection of clinically relevant gene rearrangements such as those involving in *ALK*, *ROS1*, *RET*, and *PDGFRA*.

In recent years, an increasing number of publications have reported both the potential and the limitations of the NGS application in cancer detection and diagnosis^22^. However, before NGS technologies can be applied to routine pathology molecular diagnostics, more studies are needed to ensure consistent and reliable performance. Most importantly, comparisons with current conventional tests in all aspects, such as turnaround time, sensitivity, specificity, mutation detection limits, costs, and capacity are required. In the present study, we systematically investigated the feasibility and reliability of an NGS platform for use in a routine setting using a combination of cell lines and 119 clinical samples. It is crucial to ensure the quality of the result when it may be used to guide treatment decisions. The NGS method detected all expected variations (n = 61) in samples with conditional golden methods (ARMS-PCR/FISH). The NGS platform also detected mutations that are not assayed by genotyping or allele-specific PCR-based mutation profiling platforms. Thus, NGS can detect a broader range of genomic alterations than current clinical assays which may uncover more actionable options for patients. The experimental sensitivity of our assay was estimated to be 1% and 5% for SNVs and InDels respectively, which is higher than that of routine molecular methods. Several actionable somatic alterations were detected in samples with tumour purity as low as 10–20%, which is not achievable by conventional methods (Table S2). This high sensitivity is especially important for the study of *EGFR* T790M heterogeneity, because quantitative assessment by targeted NGS could enable early predication of acquired resistance to tyrosine-kinase inhibitors (TKIs) as shown in case #A44(Table S2).

Turnaround time (TAT) and cost are key issues for the transfer of NGS technology to routine molecular diagnostics. The overall cost, including chemicals, labour, and depreciation expenses for our target NGS approach was $198 per sample, whereas the cost of Sanger sequencing for 15 genes in our lab was $647 per sample. Although the price of a total NGS run is relatively high, hybridisation based DNA enrichment and DNA barcoding decreases the sequencing cost per sample greatly. Benefiting from the fast sequencing of the Ion Proton instrument and the automatic analysis and report software, the turnaround time for the entire assay from specimen acquisition to clinical reporting is only six days, faster than the Illumina Hiseq platform (an average of five weeks) and slightly slower than the Sanger sequencing (three days)^23^.

In conclusion, our study proves the feasibility of targeted NGS for profiling of actionable genetic alterations in FFPE tumour samples. Compared with conventional gene-specific assays NGS showed great advantages in terms of higher sensitivity, lower costs per sample, a broader range of detectable mutations, and we therefore believe that NGS technologies will likely to become routine for clinical tumour sample testing in the future.

## Methods

### Tumour tissue and cell lines

This study was approved by the Institutional Review Board of Beijing Genomics Institution (BGI) and was carried out in accordance with the approved guidelines. Before the surgery, all the patients were informed about the purpose of the study and agreed by signing an informed consent. Human male genomic DNA from the YH^24^ cell line was used as a wild-type control. We studied 119 formalin fixed, paraffin-embedded tumour specimens from lung adenocarcinoma patients treated at The First Affiliated Hospital of Guangzhou Medical University (Table 5 and Table 6). All selected patients had a clinical indication for *EGFR* mutation testing. Tumour cell proportion of the specimens, reviewed by a pathologist, ranged from 10% to over 80%. Cell line genomic DNA was purchased from the American Type Culture Collection (ATCC). Confirmation of cell line genomic DNA was performed by allele-specific PCR and Sanger sequencing. Mutations detected by NGS that were not covered by the ARMS-PCR and FISH assay were confirmed by Sanger sequencing.

### DNA extraction

DNA was extracted from unstrained FFPE resections using the QIAamp DNA FFPE Tissue Kit. Xylene was added twice or more to each sample as requred for paraffin removal. Ethanol (100%) was added once for xylene removal. Samples were resuspended in Buffer ATL and proteinase K for tissue lysis at 50 °C with 800-rpm shaking until tissue were completely lysed. After 1h of 90 °C incubation, buffer AL and 100% ethanol was added for further cell lysis and DNA precipitation. DNA was eluted in 750–100 μl of Buffer ATE. The concentration of DNA was measured using a Qubit fluorometer (Life Technologies, Thermo Fisher). Agarose gel (2%) electrophoresis was performed for quality control.

### Selection of targeted genes

The chip was designed and synthesised domestically and began with including whole exons of potential driver genes related to lung cancer literarily. We then expanded it with exons containing recurrent mutations based on the Catalogue of Somatic Mutations in Cancer (COSMIC), The Cancer Genome Atlas (TCGA) and the International Cancer Genome Consortium (ICGC) to maximise the number of patients covered and the number of missense mutations per patients. Finally, introns and exons spanning recurrent fusion breakpoints of *ALK*, *ROS1*, *RET*, and *PDGFRA* were also included. Collectively, the chip targets 436 exons, 13 introns from 145 tumour related genes, in a total size of 249 kb and covers 55,984 patients’ samples from the COSMIC database.

### Hybrid selection and sequencing

Isolated FFPE DNA (200–500 ng) was fragmented prior to library construction using the Bioruptor Pico sonicator (Diagenode). After end-repairing and ligating to barcode adapters, ligated fragments were amplified for 12 cycles using Phusion High-Fidelity DNA Polymerase (Finnzymes, Thermo Fisher). Double-step size selection using Agencourt Ampure XP beads (Beckman-Coulter) was performed by adding 0.9× and 0.15× of beads to obtain fragments between 230–270 bp in length. Indexed libraries were then subjected to self-synthesised custom capture probe hybridisation. Four to six indexed libraries were pooled in a single capture hybridization at 65 °C for 24 h. After stringent washing, captured fragments were amplified for 10 cycles using Phusion High-Fidelity DNA Polymerase. Quality and quantity were measured using the Qubit fluorometer and Agilent 2100 Bioanalyzer (Agilent). Multiplexed libraries were pooled proportionally and sequenced using an Ion Proton Sequencer (Life Technologies, Thermo Fisher).

### Mutation detection and reporting

We developed a bioinformatics pipeline for processing the sequencing data named Otype in which SNVs, InDels, CNVs, and gene rearrangements are simultaneously detected with high sensitivity and specificity (Fig. 1). Raw data from Torrent Suite were processed with standard steps, which included trimming bases of low quality, alignment of reads to a reference genome hg19, and marking of duplicate reads, to get standard bam files. SNV and InDel are treated simultaneously and equally. For actionable and druggable mutations, we have archival a program which is capable of reaching sensitivity and specificity beyond 99% for mutant allelic frequencies >0.03 (supplemental materials) with Q30 reads. CNVs were detected using CONTRA, and gene arrangement is detected by SeekSV. Without a matched normal, interpretation focused on known somatic hotspot mutations, including SNVs, InDels, as well as known CNVs and gene arrangements in lung cancer. The mutations were annotated according to databases of established and experimental therapies to identify potential clinical actionability and predisposing alterations. Finally, reporting was focused on alterations associated with clinically available targeted treatment options.

### Confirmation of mutations by Sanger sequencing

We selected samples with novel mutations from targeted NGS for validation testing by allele-specific PCR and Sanger sequencing. Samples with inconsistent analysis result between targeted NGS and ARRMS/FISH were verified by Sanger sequencing. Sanger sequencing was performed using the BigDye Terminator Version v3.1 Cycle-sequencing Kit (Applied Biosystems) and an ABI 3730xl DNA sequencer (Applied Biosystems).

## Additional Information

**How to cite this article**: Shao, D. *et al*. A targeted next-generation sequencing method for identifying clinically relevant mutation profiles in lung adenocarcinoma. *Sci. Rep*. **6**, 22338; doi: 10.1038/srep22338 (2016).

## Supplementary Material

Supplementary Information

## Figures and Tables

**Table 1:** Detection limit of the targeted NGS method.

Mutation Type	Mutation Frequency	No. of Samples	Detected by NGS
SNV *EGFR* T790M	1%	**4**	**3**
	3%	3	3
	5%	8	8
	10%	3	3
SNV *EGFR* L858R	1%	**4**	**3**
	3%	3	3
	5%	8	8
	10%	3	3
InDel *EGFR* c.2235_2249del15	1%	4	4
	3%	3	3
	5%	8	8
	**10%**	**3**	**3**

**Table 2:** Intra-run reproducibility.

Mutation	barcode samples								mean	SE	Sensitivity (%)
	1	2	3	4	5	6	7	8			
Variant frequency (%)											
*EGFR* L858R	7.50%	7.30%	5.30%	7.00%	5.70%	8.80%	11.50%	5.80%	7.36%	0.019	100%
*EGFR* T790M	6.70%	4.90%	7.80%	7.50%	7.00%	11.30%	9.50%	11.90%	8.33%	0.022	100%
*EGFR* EX19 Del	5.61%	6.04%	8.57%	8.11%	8.03%	7.96%	8.36%	5.36%	7.25%	0.013	100%
Coverage(x)											
*EGFR* L858R	313	262	334	91	260	443	304	330	292.13	92.816	
*EGFR* T790M	254	178	271	91	213	333	233	226	224.88	66.139	
*EGFR* EX19 Del	410	530	992	814	872	754	825	784	747.63	176.173	

**Table 3:** Concordance of targeted NGS with conventional platforms.

Mutation Type	Gene	Platforms	Expected	Detected	% Concordance
SNV	*EGFR*	ARMS-PCR	22	22	100%
SNV	*KRAS*	ARMS-PCR	16	16	100%
SNV	*PIK3CA*	ARMS-PCR	2	2	100%
Deletion	*EGFR*	ARMS-PCR	16	16	100%
Insertion	*EGFR*	ARMS-PCR	1	1	100%
Gene Fusion	*ROS1*	FISH	2	2	100%
Gene Fusion	*ALK*	FISH	1	1	100%
Gene Fusion	*RET*	FISH	1	1	100%
Total	–	–	61	61	100%

**Table 4:** Variant classification scheme.

Pattern	Level	Evidence
Sensitivity to targeted drugs	1A	Drug is FDA-approved for lung cancers harbouring the variant.
	1B	Drug is clinically effective in the biomarker-selected cohort of lung cancer. The variant, which has a definite function feature, belongs to the cohort.
	2A	Drug is FDA-approved for another tumour type harbouring the variant. Clinical trials have proved its effectiveness in lung cancer patients with the variant.
	2B	Drug is FDA-approved for another tumour type harbouring the variant. Case reports have indicated its effectiveness in lung cancer patients with the variant.
	2C	Drug is FDA-approved for another tumour type harbouring the variant. Pre-clinical research has indicated its effectiveness in lung cancer patients with the variant.
Resistance to targeted drugs	1A	NCCN guideline has clearly indicated that the variant is associated with resistance to the FDA-approved lung cancer drug.
	1B	NCCN guideline has indicated drug resistance to the biomarker-selected cohort of lung cancer. The variant, which has a definite function feature, belongs to the cohort.
	2A	Case reports have indicated drug resistance in the lung cancer patients with the variant.
	2B	Pre-clinical research has indicated drug resistance in lung cancer patients with the variant.

**Table 5:** Demographic and histopathological features of the archived samples.

Characteristic	N = 61	%
Gender		
Male	26	42.6
Female	35	57.4
Age		
Average	63.9	–
Range	41–88	–
Smoker		
Never	40	65.6
Light (pack-year < 30)	8	13.1
Heavy (pack-year > 30)	13	21.3
Grade		
G1	11	18.0
G2	44	72.1
G3	6	9.8
Stage		
I	13	21.3
II	21	34.4
III	19	31.1
V	8	13.1
Tumour content		
10%–30%	31	50.8
30%–70%	28	45.9
>70%	2	3.3

**Table 6:** Demographic and histopathological features of the prospective samples.

Characteristic	N = 58	%
Gender		
Male	28	48.3
Female	30	51.7
Age		
Average	61.9	–
Range	38–81	–
Smoker		
Never	33	56.9
Light (pack-year <30)	5	8.6
Heavy (pack-year >30)	20	34.5
Grade		
G1	13	22.4
G2	38	65.5
G3	7	12.1
Stage		
I	21	36.2
II	13	22.4
III	17	29.3
V	7	12.1
Tumour content		
10%–30%	31	53.4
30%–70%	25	43.1
>70%	2	3.4
