# Supplementary material for: A targeted next-generation sequencing method for identifying clinically relevant mutation profiles in lung adenocarcinoma
Source: Sci Rep. 2016 Mar 3;6:22338. doi: 10.1038/srep22338 (PMC4776238; doi:10.1038/srep22338)
Supplement: Supplementary Information [file srep22338-s1.pdf]

Title

A targeted next-generation sequencing method for identifying clinically relevant mutation profiles in lung adenocarcinoma

Author and affiliation

Di Shao[1,2,3*], Yongping Lin[4,5,6*], Jilong Liu[1], Liang Wan[1], Zu Liu[1], Shaomin Cheng[1], Lingna Fei[1], Rongqing Deng[1], Jian Wang[1], Xi Chen[1], Liping Liu[6], Xia Gu[6] , Wenhua Liang[6], Ping He[6], Jun Wang[2], Mingzhi Ye [1,2,3]†, Jianxing He[6]†
1 BGI-Shenzhen, Shenzhen, 518083, China
2 Department of Biology, University of Copenhagen, Copenhagen, DK-2200, Denmark
3 BGI-Guangzhou, Guangzhou Key Laboratory of Cancer Trans-Omics Research, Guangzhou, 510006, China
4 Department of Laboratory Medicine, The First Affiliated Hospital of Guangzhou Medical University, Guangzhou, 510120, China
5 Centre of Translational Medicine, The First Affiliated Hospital of Guangzhou Medical University, Guangzhou, 510120, China
6 The First Affiliated Hospital of Guangzhou Medical University, Guangzhou, 510120, China
*These authors contribute equally to the work
†Correspondence should be addressed to hejx@vip.163.com and yemingzhi@genomics.cn

Supplementary Information

Table S1 targeted gene list

**PART I: Actionable now or in future**	**PARTII: Recurrent in database**	
	**COSMIC**	**TCGA / ICGC**
AKT1,	*CDKN2A, STK11, KEAP1, PDGFRA, KLK1,*	*ZFHX4, ZNF804A, DCAF4L2,*
EGFR,	*NFE2L2, CTNNB1, SMAD4, FGFR2, APC,*	*OR5D18, ZNF479, OR51V1,*
KRAS,	*FBXW7, HRAS, ERBB4, MUC6, CHEK2,*	*OR4N2, OR4C15, OR14C36,*
NRAS,	*NBPF10, PARG, FBN2, HSD17B7P2, NF1,*	*CROCC, OR2T2, PCDH11X,*
BRAF,	*WASH2P, POTEC, EEF1B2, ADAM23, TP53,*	*REG3A, REG1B, DDX11,*
FGFR1,	*DNMT3B, SDHAP2, DHX9, CSNK2A1, KDR,*	*DNAH8, OR2B11, OR4K2,*
PIK3CA,	*CNTN5, ATXN3, CLIP1, OR4M2, NOTCH1,*	*FAM47A, PRB2, CNTNAP2,*
DDR2,	*OR10G8, PAPPA2, OR8H2, PBX2, FGFR3,*	*CDH10, CDH12, RALGAPB,*
NTRK1,	*POLDIP2, SLC6A10P, ZNF804B, ZEB1, RB1,*	*LRRIQ3, OR5L2, OR2T33,*
PTEN,	*GAB1, OR10Z1, CNTNAP3B, XIRP2, JAK2,*	*RYR2, EPB41L4A, OR2M2,*
HER2,	*KIAA0907, IL32, TNRC6A, FGFR4, DDX11L2,*	*OR4C16, KCNB2, STAG3L2,*
ALK,	*FAM135B, VGLL3, NYAP2, TSHZ3, NTRK3,*	*OR2T34, LPA, MMP27,*
ROS1,	*ANAPC1, FAM47C, AKAP6, DSPP, FRG1B,*	*VAV3, THSD4, OR4N4,*

RET,<br>*MET*	*KRTAP5-5, ATXN1, MUC16, BEST3, KIT, KRTAP4-11, ZNF814, EPHA5, EPHA3, ATM, NUDT11, SNAPC4, ZNF598, PTPRD, INHBA, KIAA2022, MB21D2, MAP1B, NAV3, JAK3*	*NOTCH2, FOLH1, GNA15, KRTAP4-8, TBX6, WDR62*

Table S2. Validation of with known mutations in 61 archival samples

No.	Mutation Type	Clinical Result	Detected by NGS	Coverage(X)	Mutation Frequency
A1	SNV	*EGFR* G719S	*EGFR* G719S	224	36.6%
A2*	SNV	*EGFR* S768I	*EGFR* S768I	878	7.6%
A3	Del.	*EGFR* EX19 Del.	*EGFR* EX19 Del.	225	22.2%
A4	Del.	*EGFR* EX19 Del.	*EGFR* EX19 Del.	531	30.1%
A5	Del.	*EGFR* EX19 Del.	*EGFR* EX19 Del.	375	16.3%
A6	Del.	*EGFR* EX19 Del.	*EGFR* EX19 Del.	523	26.2%
A7	Ins.	*EGFR* EX20 Ins.	*EGFR* EX20 Ins.	774	52.5%
A8	SNV	*EGFR* L861Q	*EGFR* L861Q	891	26.3%
A9	SNV	*EGFR* L861Q	*EGFR* L861Q	754	13.6%
A10	SNV	*EGFR* L858R	*EGFR* L858R	537	35.2%
A11	SNV	*EGFR* L858R	*EGFR* L858R	995	33.9%
A12	SNV	*EGFR* L858R	*EGFR* L858R	1008	38.7%
A13	SNV	*EGFR* L858R	*EGFR* L858R	1467	30.6%
A14	SNV	*KRAS* G12D	*KRAS* G12D	288	34.7%
A15	SNV	*KRAS* G12D	*KRAS* G12D	365	29.3%
A16	SNV	*KRAS* G12V	*KRAS* G12V	380	46.6%
A17	Fusion	*ROS1* fusion	*ROS1* fusion	--	--
A18	Fusion	*ROS1* fusion	*ROS1* fusion	--	--
A19	SNV	*PIK3CA* E545D	*PIK3CA* E545D/*KRAS* G12D	313/313	27.15%/27.15%
A20	SNV	*PIK3CA* H1047R	*PIK3CA* H1047R	152	9.9%
A21	Del.	*EGFR* EX19 Del.	*EGFR* EX19 Del./*PIK3CA* H1047L	602/433	37.54%/14.87%

A22	Del.	*EGFR* EX19 Del.	*EGFR* EX19 Del.	805	30.4%
A23	Del.	*EGFR* EX19 Del.	*EGFR* EX19 Del.	523	34.1%
A24	Del.	*EGFR* EX19 Del.	*EGFR* EX19 Del.	828	14.1%
A25*	Del.	*EGFR* EX19 Del.	*EGFR* EX19 Del.	668	9.4%
A26	Del.	*EGFR* EX19 Del.	*EGFR* EX19 Del.	815	12.5%
A27	Del.	*EGFR* EX19 Del.	*EGFR* EX19 Del.	951	20.7%
A28*	Del.	*EGFR* EX19 Del.	*EGFR* EX19 Del.	607	8.1%
A29*	Del.	*EGFR* EX19 Del.	*EGFR* EX19 Del.	480	9.0%
A30	Del.	*EGFR* EX19 Del.	*EGFR* EX19 Del.	648	21.0%
A31	Del.	*EGFR* EX19 Del.	*EGFR* EX19 Del.	712	21.5%
A32	SNV	*EGFR* L858R	*EGFR* L858R	940	29.0%
A33	SNV	*EGFR* L858R	*EGFR* L858R	1268	14.0%
A34	SNV	*EGFR* L858R	*EGFR* L858R	912	19.5%
A35	SNV	*EGFR* L858R	*EGFR* L858R	901	13.5%
A36	SNV	*EGFR* L858R	*EGFR* L858R	831	26.4%
A37	SNV	*EGFR* L858R	*EGFR* L858R	971	14.3%
A38	SNV	*EGFR* L858R	*EGFR* L858R	1246	19.7%
A39*	SNV	*EGFR* L858R	*EGFR* L858R	738	15.3%
A40	SNV	*EGFR* L858R	*EGFR* L858R	723	46.9%
A41	SNV	*EGFR* L858R	*EGFR* L858R	422	23.5%
A42	SNV	*EGFR* L858R	*EGFR* L858R	591	23.0%
A43	SNV	*EGFR* L858R	*EGFR* L858R	487	30.4%
A44	SNV	*EGFR* L858R/ *EGFR* T790M	*EGFR* L858R/ *EGFR* T790M	1696/2449	54.48%/56.96 %
A45	SNV	*EGFR* L861Q	*EGFR* L861Q	1520	23.1%
A46	SNV	*KRAS* G12C	*KRAS* G12C /*NRAS* Q61K	356//396	6.46%/11.11%
A47*	SNV	*KRAS* G12C	*KRAS* G12C	463	12.1%
A48	SNV	*KRAS* G12C	*KRAS* G12C	445	36.2%
A49	SNV	*KRAS* G12C	*KRAS* G12C	392	26.8%
A50	SNV	*KRAS* G12C	*KRAS* G12C	409	17.4%
A51	SNV	*KRAS* G12C	*KRAS* G12C	535	23.6%
A52	SNV	*KRAS* G12D	*KRAS* G12D	387	20.4%
A53	SNV	*KRAS* G12D	*KRAS* G12D	244	41.4%
A54	SNV	*KRAS* G12D	*KRAS* G12D	225	28.0%
A55*	SNV	*KRAS* G12V	*KRAS* G12V	386	13.2%
A56	SNV	*KRAS* G12V	*KRAS* G12V	305	21.3%
A57	SNV	*KRAS* G12V	*KRAS* G12V	333	28.8%
A58	SNV	*KRAS* G12V	*KRAS* G12V	300	17.7%
A59	Fusion	*ALK*++	*EML4/ALK*	--	--
A60	Del.	*EGFR* EX19 Del.	*EGFR* EX19 Del.	1397	18.5%
A61	Fusion	*RET* fusion	*RET* fusion	--	--

* Targeted NGS detected mutations in samples containing only 10% to 15% tumor cells

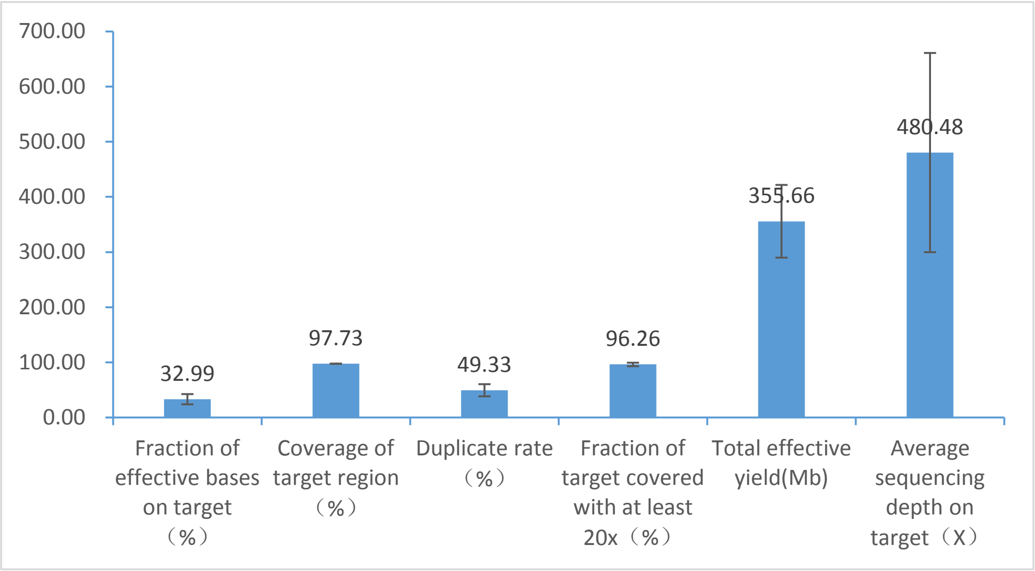

Figure S1. Capture performance of 119 clinical FFPE samples

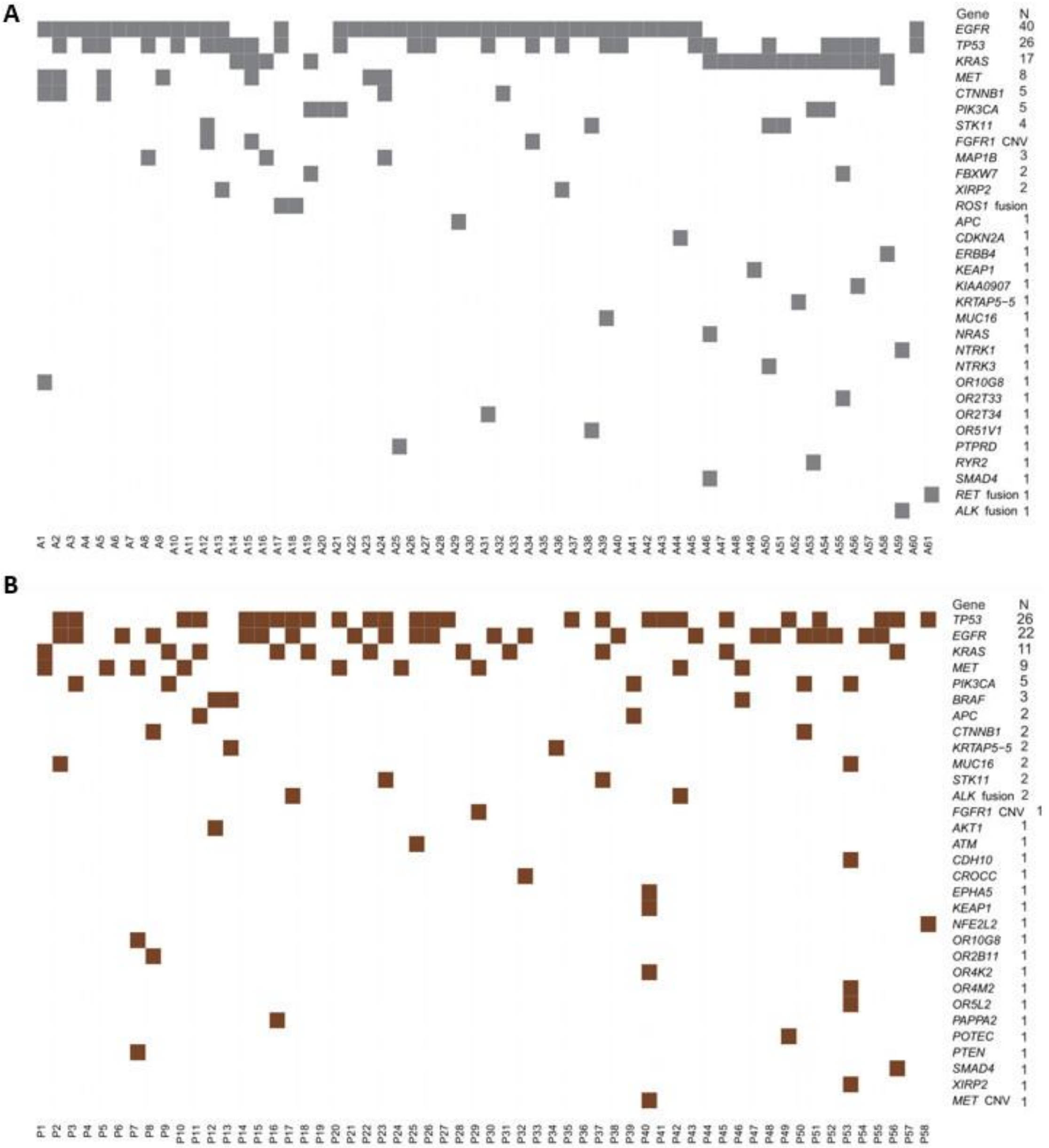

Figure S2. Somatic mutations identified in archival samples (A) and prospective samples (B). Each column represents 1 sample; each row represents 1 gene. The column on the right (n) indicates the number of samples with specific gene mutation
